# Addressing unforeseen public health risks via the use of sustainable system and process management

**DOI:** 10.3389/fpubh.2023.1249277

**Published:** 2023-11-10

**Authors:** Yi Zhang, Yue Yu, Patrick Sik Wah Fong, Jianfu Shen

**Affiliations:** ^1^Department of Building and Real Estate, The Hong Kong Polytechnic University, Hong Kong, China; ^2^Department of Land Surveying and Geo-Informatics, The Hong Kong Polytechnic University, Hong Kong, China; ^3^School of Engineering and Built Environment, Griffith University, Brisbane, QLD, Australia

**Keywords:** epidemics, unknown risk, prevention system, loose coupling, sustainability

## Abstract

During the coronavirus disease 2019 (COVID-19) pandemic, the severe acute respiratory syndrome coronavirus 2 (SARS-CoV-2), which was designated by the World Health Organization in January 2020 as a newly emerging coronavirus in 2019, and its variants have placed unbearable strain on the healthcare systems of various countries, with serious implications for sustainable development worldwide. Researchers have proposed several solutions, such as the use of digital technologies to improve prevention systems. However, the challenges of epidemic prevention and control failures have not been addressed fundamentally, as the key causes of epidemic failures (i.e., outbreaks) and strategies for process management have been neglected. The purpose of the current study is to address these issues by exploring the causes of epidemic prevention and control failure and targeting improvement strategies that combine system structure of epidemic prevention and process management. Specifically, following an exploration of the main reasons for COVID-19 prevention and control failures through a case study of two tertiary hospitals, this paper outlines a targeted prevention and control system based on triangular validation and a loosely coupled process management framework and verifies the expected results using simulation methods together with statistical data on the spread of SARS-CoV-2 in Wuhan, China. The findings not only advance the development of epidemic risk prevention and control theory, especially the complementary nature of IT applications and process management in the field of epidemic risk prevention and control, but also provide guidance on the innovation and implementation of epidemic prevention and control systems and process management and recommendations for countries to promote sustainable development from a health-focused perspective.

## 1. Introduction

Since early 2020, the sudden onslaught and rapid spread of severe acute respiratory syndrome-coronavirus 2 (SARS-CoV-2) and the subsequent coronavirus 2019 (COVID-19) pandemic have paralyzed the world and temporarily caused global sustainable development to come to a standstill. According to the 2021 United Nations Sustainable Development Goals report^1^, COVID-19 has had a catastrophic global impact. For example, the number of people living in poverty has increased from 119 million to 124 million, 255 million full-time jobs have been destroyed, and the basic survival of 1.6 billion informal workers have been jeopardized. The global COVID-19 pandemic has spread to more than 170 countries, infecting more than 651 million people and causing approximately 6.6 million deaths until 23 of December, 2022.

The rapid escalation of the COVID-19 pandemic has clearly exposed the inability of some countries to respond to epidemic risks, especially in terms of the responsiveness of their outbreak prevention and control systems and corresponding process management. For example, although early cases of SARS-CoV-2 infection were reported in Wuhan, China, the city was not closed until January 24, 2023, causing the government to miss a 14-day golden period for epidemic prevention and control. Accordingly, this delay led to a geometric increase in patients with COVID-19. India faced a similarly desperate situation associated with an outbreak of infection with the Alpha variant of SARS-CoV-2, and South Africa’s prevention and control system was unable to cope with the spread of the Beta variant. However, these countries had previously implemented effective early warning systems in response to sudden outbreaks of H1N1 influenza, Middle East respiratory syndrome (MERS) and Ebola hemorrhagic fever (EBHF). Therefore, countries around the world are focusing on the reasons underlying the failure of COVID-19 prevention and control measures and making corresponding changes to strengthen their capacity to respond to similar pandemic risks in future emergency situations and further promote sustainable development.

One fundamental reason that has been proposed to explain the observed failures in pandemic control is the pathogenic features of SARS-CoV-2, which vary from those of viruses associated with prior epidemics. These differences in pathogenic features have led to distinct virus risk profiles. The origins of risk may be traced back to research reported by Knight (1), in which risk was found to be coupled with uncertainty. Risk can be divided into known and unknown risks according to the knowledge hierarchy (i.e., level of understanding) (2). Statistical estimation indicates that approximately 4,000 viruses exist in nature, 95% of which are unknown to humans. Among the 5% of viruses known to humans, coronaviruses received little attention until the late 1990s and have remained the focus of a vaguely localized area of study for nearly 20 years. In other words, the study of viruses is rife with unknowns, which may enable outbreaks of epidemic diseases that cannot be anticipated by existing knowledge governing prevention systems (3). According to Hagigi and Sivakumar (4), risk can be classified as born or unborn, depending on whether the risk is associated with an outbreak or widespread transmission. For example, India regarded the original strain of SARS-CoV-2 as a born risk but the Alpha variant as an unborn risk. Thus, unknown or known risk was much related to the time which means that risk has been happened in the same area or not; while born or unborn was much related to area, means that a risk has been happened in other area during the same period Most existing epidemiological methods for outbreak control are well suited to cope with born-known, unborn-known, and born-unknown risks but struggle to handle unborn-unknown risks. In addition, although studies have plausibly explained the risk factors for epidemic disease outbreaks, they have not provided precise methods for controlling each factor.

Lisa Gralinski, an associate researcher in Ralph Baric’s laboratory in North Carolina, United States, emphasized the importance of preventive technology in countering epidemic outbreaks. However, clinical presentations are heterogeneous, and the early diagnosis of epidemic diseases is uncertain; consequently, reliable information must be extracted from both structured and unstructured data, and this need has increased the requirement for analytical skill with digital technologies (5, 6). The use of new digital technologies, such as big data and artificial intelligence, has been proposed to expand the scope of surveillance in prevention systems, unify data structures to facilitate information sharing, and connect internal pathways within healthcare systems to facilitate information transfer (7–9). Additionally, some experts have stressed the value of combining digital technologies with social media data to enhance predictive information (10, 11). Although these studies have successfully fostered the enhancement of outbreak prevention technology, several flaws remain. For instance, data collection in the context of prevention has been limited to social media users, and data from third parties such as academic research institutes have been neglected. Furthermore, SARS-CoV-2 has spread more rapidly and behaved more unpredictably than other pathogens that have caused epidemic outbreaks and thus requires a capacity for rapid process reaction. To date, however, research has not provided comprehensive suggestions for strengthening prevention systems while simultaneously increasing response capabilities.

A few scholars have suggested that hospitals’ established process management are characterized by features such as cascading reports, centralized aggregation, and centralized surveys; these lead to an inherent path dependency and make it difficult for prevention systems or countries to implement timely reassessment strategies or resilient responses to rapidly control the spread of unborn-unknown risks (12, 13). Others have suggested that the low probability of an outbreak (14) frequently results in a disregard for prevention management, especially process management, due to the low urgency associated with the risk probability, and this disregard can lead to a deficiency in process management. Additionally, the insufficient understanding, attitudes, and behaviors of healthcare workers with respect to the control systems used in prevention and control efforts might increase the risks (15). In summary, the research has highlighted the difficulties associated with process management practices and the elements that drive them but has not shed light on their bottom-up process management preventive strategies or presented solutions.

To address research issues in the failure of epidemic prevention and control, the study described in this paper uses theoretical risk types to investigate the causes of failure to prevent outbreaks of unborn-unknown risks in both the technical and process dimensions. A triangulated validation system based on big data and artificial intelligence technologies is proposed, and a loosely coupled process management framework is extended. This paper also includes an analysis of the expected results. Conclusions can be drawn via simulation analysis and interview-based case studies, with great theoretical and practical significance for countries aiming to strengthen their risk prevention systems and emergency management processes.

## 2. Literature review

The fundamental academic rationale of this study is set on the following premises: first, the prevention and control of epidemic outbreaks are crucial elements of sustainable development; second, the causes for, and preventative measures against, varying risk categories of epidemics must be differentiated; third, tackling epidemic outbreaks involves enhancing the direct reporting system, particularly through data structure adjustments and improvements; fourth, adaptive process management forms the core of an organization’s ability to respond swiftly to rapid environmental changes, thereby better equipping them to handle pandemic situations. Consequently, our literature review is structured into four key sections: Sustainability and Health; Uncertainty and Health Risk; Systems Technology and Adaptive Process Management and Loose Coupling; and Decision-Making Frameworks.

Our study specifically dwells on the terms “Sustainability,” “Uncertainty,” “Risk,” “Systems Technology,” “Health Risk,” “Adaptive Process Management,” and “Decision-Making.” We identified literature pertinent to our research by using these keywords in Web of Science searches. Given that our paper fundamentally pertains to the fields of management and preventative medicine, we streamlined the disciplines to “Preventive and Control Medicine” and “Management.” Also, we confined our selection to articles published between 2012 and 2022. An initial selection yielded 1,044 articles. Following preliminary analysis of the titles and profiles of these papers, we hand-picked 68 of the most relevant for further reference.

### 2.1. Sustainability and health

The term “sustainable development” has gained prominence in the last decade (16). Sustainable development encompasses economic, societal, and environmental considerations. However, it has been proposed (17) that sustainable development extends into health, drawing particular attention to how health improvements are integrally connected to at least ten of the Sustainable Development Goals (SDGs). These include eradicating poverty and inequality, promoting health and well-being, and safeguarding the environment to ensure sustainable growth. Hence, sustainable health progress is an integral international public policy issue for achieving sustainable development (18).

China has underscored its most considerable health sustainability challenges, including increasing epidemics (19). It has been noted that pandemic responses are the most crucial types of health interventions in programs relating to disease prevention and health promotion (20). A sustainable health system capable of responding to a pandemic should possess three key attributes: affordability for patients and families, employers, and governments; acceptability for key populations such as patients and health professionals; and an adaptive response to an unexpected pandemic (21).

Enhancing the efficiency of health case prevention and control systems may not be an easy task. Some research has delved into enhancing the efficiency of these systems from a technological angle (22, 23). However, such research usually targets a single dimension, namely the effectiveness of the prevention and control systems, with scant attention given to the merging of process management and systemic change dimensions. Therefore, it becomes pivotal to amalgamate both administrative and systemic perspectives to ramp up disease prevention odds.

### 2.2. Uncertainty and health risk

Research on uncertainty can be traced back as far as 1921, when (1) categorized uncertainty into quantifiable and non-quantifiable forms. A quantifiable form was identified as a type of uncertainty that can be reduced to a finite probability distribution with sufficient information and knowledge; i.e., it can be transformed into a known risk. A non-quantifiable form was identified as uncertainty that cannot be quantified in advance and is difficult to transform into a manageable risk through data analysis and knowledge acquisition due to its unknown nature.

Known risks have always been central to risk prevention and management concerns related to the prevention and control of pandemic outbreaks (24). In contrast, unknown risks frequently present challenges and become weak points in prevention and control efforts due to the variability of disease symptoms, diversity of causes, extent of spread, and complexity of related hazards (25). First, unknown risks often exhibit irregular clinical variation because of their low prevalence and spread and thus are difficult to identify in the early stage based on patients’ symptoms. Healthcare workers are constrained by time and attention (5) and frequently find it difficult to deduce novel causal relationships in the short term, instead relying on established knowledge and experience to produce unclear explanations (14). Second, the initial clinical features of an unknown risk are often similar to those of a known risk. For example, COVID-19 has a median incubation period of 3 days (26), and its initial symptoms include malaise, cough, mild fever, and even changes in blood count in certain individuals, making it difficult to detect a sudden outbreaks at the early stage. Third, the range of possible causes hinders healthcare personnel’s ability to determine the source of illness. This makes it difficult to characterize epidemic diseases and their transmission routes (27).

If epidemic breakouts or transmission networks at the national or regional level are evaluated as a system, the risk of an epidemic may be classified as born or unborn (4). For example, in China, SARS and SARS-CoV-2 were initially concentrated and widely spread within the nation, necessitating a proactive response in terms the nation’s own attitudes, systems, and technologies for epidemic preparedness, and thus were regarded as unborn risks. For other external parties, such risks are regarded as born risks, as the outbreak is known but has not yet spread to their system. For example, the initial outbreak of the Alpha variant of SARS-CoV-2 occurred in India, and hence was considered an unborn risk for that country, whereas it was a born risk for other nations such as China, the United States, and Italy.

In summary, risks can be classified as known or unknown according to the level of relevant knowledge, or as born or unborn according to differences in the outbreak circumstances. These classifications yield a 2 × 2 classification framework to describe four types of risk, namely unborn-known, born-known, born-unknown, and unborn-unknown. For example, in China, unborn-known risks include diseases such as hepatitis B or tuberculosis; born-known risks include, for example, EBHF; born-unknown risks include diseases such as MERS; and unborn-unknown risks include, for example, SARS and COVID-19.

### 2.3. Systems technology

Compared with known risks for which the pathogen, transmission routes, and outbreak conditions are known, an unknown risk is associated with uncertainty; such a risk is difficult to translate into a controllable risk and thus resembles a “black swan” event (28). Currently, there are two primary strategies for dealing with such unknown risks. First, technological advancements can increase the availability of knowledge about the risk to enable early prediction and judgment (2). This strategy entails the examination of system and media data. For example, multidimensional big data, such as personal sleep and body temperature, population movement tracking, and social media sentiment, can be leveraged (29). Second, institutional change can maximize the separation of risks from unknown risks. This type of strategy involves a quick and flexible response to the risk components that can be isolated and controlled during the dynamic process of a sudden epidemic. Such a strategy entails altering the associated management procedures.

Integrated information technology (IT) applications are crucial for predicting, diagnosing, and controlling epidemic disease outbreaks (30) through techniques such as time series analysis, simulation modeling, social network analysis, and geographic visualization of epidemic diseases in conjunction with their transmission characteristics to detect and determine disease development trends in real or near-real time. This information may be used to guide public health decisions for prevention and control (6). “Prevention system” is a broad term that refers to the establishment of an information monitoring and transfer platform to collect, process, store, retrieve, analyze, research, and make decisions on behalf of regional health prevention and control administrative departments at all levels (31). The fundamental information system “Web-based Direct Reporting System for Infectious Disease Outbreaks and Public Health Emergencies” for disease prevention and control consists of six subsystems, including a statutory epidemic disease system, an emergency public health event reporting system, a statutory epidemic disease surveillance system, and a statutory epidemic disease surveillance system (32). As a result, the system can establish a quick response model based on an epidemic illness and public health emergency case database. Ideally, an effective response from the Center for Disease Control (CDC) in China would be generated only 4 h after the detection of an epidemic disease at the grassroots level (33).

However, this strategy is contingent upon the comprehensive reporting of threat cases by a local health facility or the CDC: specifically, the categorization of an epidemic and the case descriptions supplied by a local health facility or the CDC serve as the foundation for a quick reaction. Generally, local health institutions or the CDC can properly categorize or completely characterize the known risks and offer enough new case data to enable the prevention system to react effectively. However, local health institutions or the CDC may be unable to precisely categorize or adequately characterize an unborn-unknown risk, thus hindering the prevention and control system’s ability to generate an effective response. There is a tremendous need to alter the data structures of current systems by utilizing IT to control such unborn-unknown risks (6).

### 2.4. Adaptive process management and loose coupling decision-making frameworks

Apart from digital technology, an information system’s value is decided by its supporting procedures (34, 35). That is, to realize value, the managers and technology users must develop trust in the information systems technology itself, while members of the organization must develop a whole process response solution driven by data, information, and the system. The following theoretical explanation has been put forth to explain the failure of systems and process management to perform as expected with respect to the prevention and control of COVID-19 in Wuhan, China: when an organization lacks awareness of the applications and extended functions of information systems, as well as the process management of their applications, data analysis can only guide managers’ decision-making as the true determinants continue to be based on individual experience (36). In an outbreak epidemic scenario, this explanation is demonstrated by the fact that although a prevention and control system exists and is equipped with special channels for a rapid response, the system is not fully triggered during the 20-day period between the concentration of an unborn-unknown epidemic and the wide spread of disease. The implemented measures are still cascaded, evaluated, and investigated centrally, demonstrating a remaining distinction between technological innovation and application management.

Research has shown that this fragmentation between information systems applications and management processes is key to a country’s inability to mount an agile response to an unborn-unknown risk. Addressing the issue of independence between technological innovation and application management in the context of risk prevention and control will require changes to management processes or organizational practices. Organizational practices are interconnected, repeatable, and recognizable norms of action and patterns of behavior that are carried out by several actors (37); one example is the process of increasing the identification of certain diseases. Organizational practices provide a crucial foundation for not only organizational activity but also everyday operations.

However, the diversity of diseases and causes makes it difficult for a control system to establish uniform diagnostic criteria for various epidemic diseases, and new organizational practices are required to integrate data and information into decision-making systems to proactively identify and rapidly search diseases through practice updates and thus adapt to rapidly changing circumstances (38). For instance, the “Koch Rule,” which requires the isolation of a pathogen or viral strain before identifying the pathogen responsible for an epidemic, may result in delayed preventive measures (39). Avoiding or mitigating the costs associated with such a situation will require both technological and process changes (40), such as the adoption of next-generation sequencing (NGS) to determine viral genome sequences and modification of the process of reporting, aggregating, and analyzing data in control systems. It is essential to understand that simply improving technology without changing process management is insufficient in terms of handling unborn-unknown or even existing born-unknown risk shocks, as IT embedded in an organization can only fully enhance the efficiency of the organization’s rapid response when its use transitions from exploratory to institutionalized (41, 42).

Adaptability is the ability to adapt effectively in response to changing conditions, with a focus on quicker and more effective iteration (43, 44). “Adaptive management” refers to organizations that overcome organizational inertia by coordinating resource allocation in response to changing demands in the external environment (45). Organizational inertia develops because of the process and institutionalization of organizational operations; excessive inertia can render an organization incapable of adapting, leading it to face survival issues (46). In this paper, “adaptive process management” refers to management processes that are dynamically aligned with an organization’s or system’s intended reactions and adjustments to four distinct types of unforeseen pandemic threats.

Adaptive process management is a proactive form of adaptive management that focuses on two critical adaptive decision-making issues: (1) how to accurately distinguish adequate prevention from overreaction, and (2) how to handle the complementarity of collective and individual decision-making to organically unify democracy and centralization in risk assessment and decision-making. Theoretically, a loose coupling decision-making framework is required for proactive adaptive management decisions. “Coupling” refers to the existence of interconnected elements within a system that maintain a degree of certainty and stability in the overall system. “Loose” means that the individual, team, subsystem, and other elements of the system have some capacity to change independently to adapt quickly to changes in the external environment (47). One example of a loosely coupled decision-making structure in China involves the Meteorological Office’s forecasts of typhoons and the government’s decisions to suspend work: the Meteorological Office makes a professional judgment about the level of warning to issue and provides risk warnings, while the government makes decisions about collective risk control actions within a multi-objective framework, which inform individuals’ or groups’ risk control actions. Using a sub-delegation strategy, this loosely connected decision-making structure preserves the basic responsiveness of forecasting while avoiding the loss of risk warning capacity through its integration into governmental multi-objective decision-making (48, 49).

Unlike known risks, unknown risks are not only defined by variability and widespread transmission but also have extremely short lead times for adaptive management. All above unknown risks are the key focal points of decisions in a loosely coupled decision-making architecture. In a situation such as the COVID-19 pandemic, misleading surveillance data, such as false negative results in certain patients, may result in misclassification and increase the risk of viral propagation. In the early stage, multiple inconsistent judgments are unavoidable and frontline healthcare workers, while intuitively equipped with field experience, struggle to collect large amounts of data or information that cannot be presented in a structured manner in the short term (50); these limitations create a decision support dilemma, with incomplete information and data provided by at the grassroots level to higher-level but non-frontline decision makers (5). Addressing the information asymmetries between decision makers and frontline workers has long been a key area for improvement in the context of loosely coupled decision-making for pandemic planning and containment (6, 51).

### 2.5. Concluding comments

In summary, the prevention and process management systems developed in response to SARS and other types of risks can generate agile responses to born-known, unborn-known, and born-unknown risks; however, such systems have not functioned as expected in the face of SARS-CoV-2 and its mutated strains and variants. From the perspective of responsiveness to the unknown risks of a pandemic outbreak, the main reasons for these failure of system function can be found mainly in the structure of the network data and the design of process management.

**Figure 1:**
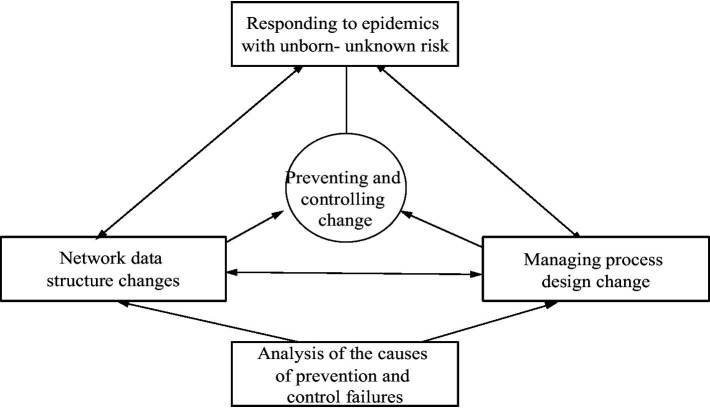
Proposed framework of a prevention system in response to an unknown risk (Source: authors’ summary).

**Figure 2:**
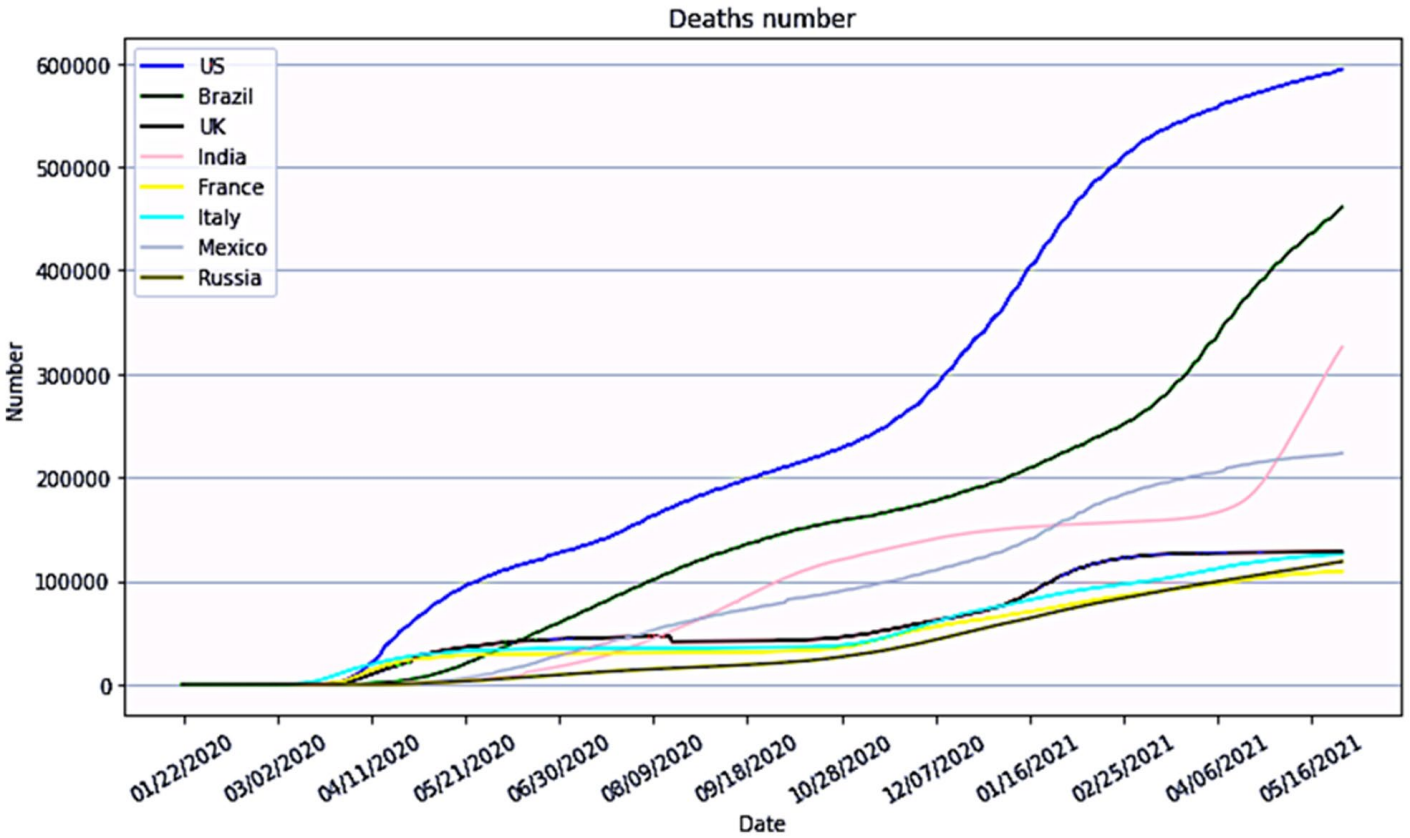
Global situation of unknown risk of major outbreaks of epidemic diseases (Source: World Health Organization).

To address this problem, this study proposes a research framework for managing change in prevention and control systems and processes for dealing with the unknown risks of pandemic outbreaks, as shown in Figure 1. The fundamental academic premise of this framework is that reacting to unknown risks enables organizations to avoid and manage changes in the unexpected environment and respond in an agile manner; accordingly, the organizations must be better prepared to cope with unknown threats (52, 53). Especially given the difficulty of predicting when unknown risks will occur and the trends and scope of impact of such risks, an agile response data structure and loosely coupled management process are effective ways to reduce information asymmetry between the decision-makers and frontline workers and represents a key direction for changes in emergency management (see Figure 2).

## 3. Method

This study is focused on changes in prevention technology and process management made to handle unborn-unknown risks. It is important to both propose changes to improve prevention and control and statically analyze the expected effects of the improvements. In particular, this study aims to, first, explore the root causes of epidemic prevention and control failures, second, base the analysis of these causes on targeted theoretical change models, and third, conduct a comparative analysis of the enhancements’ effectiveness via simulation.

Case study methodology is a highly effective tool for investigating the underlying causes of epidemic prevention and control failures due to its ability to explore key “events” impacting these failures. It allows for probing the root reasons behind these failures, bearing in mind these causes are multifaceted, encompassing both subjective and objective elements. Moreover, given the realities of prevention and control failures in China, this method offers comprehensive insight with its rich, in-depth analyses.

Following this, the study capitalizes on simulation analyses to gage the efficacy of systemic improvements in epidemic prevention and control, and in process management. Epidemic prevention and control management represents a unique area in comparison to traditional business management. It pertains to a situational entity characterized by the complexity of diverse participant interactions and is the fruition of collective societal actions, either conscious or unconscious. Given this backdrop of multi-actor interactions and collective actions, simulation analyses render meticulously detailed portrayals of expected enhancement outcomes.

In summation, the amalgamation of simulation analysis and case study methodology is a fulsome research approach for this study. Firstly, it traces the failure of epidemic prevention and control to the dual dimensions of system and process management. Secondly, targeted remedial measures are identified for these failures, underscored by detailed explanations of the triangular validation system and loosely coupled process management system designs. Lastly, the applicability of proposed improvement strategies is examined through simulation analysis.

### 3.1. Case study

#### 3.1.1. Sampling principles and data collection

**Figure 3:**
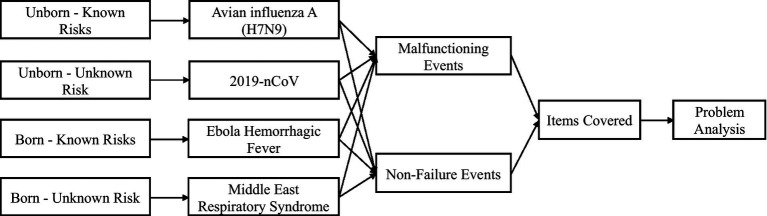
Four risk types and the key event analysis framework of this study (Source: authors’ summary).

H7N9 avian influenza A, MERS, EBHF, and COVID-19 were selected as the unborn-known, born-unknown, born-known risk, and unborn-unknown risks, respectively. The chosen diseases are characteristically the best representatives of the four risk types in terms of answering the research questions posed in this paper. First, there are similarities and differences between the four typical risks: EBHF and MERS are born risks, and H7N9 avian influenza A and COVID-19 are unborn risks; MERS and COVID-19 are unknown risks, while EBHF and H7N9 avian influenza A are known risks; accordingly, these four diseases fit well with the research questions in this paper (as shown in Figure 3). Second, the failures of prevention and control systems and process management, and the items causing these failures, differ between the four risk types, providing a basis for comparative analysis. For the purposes of this paper, “failure” refers to a failure of a system and its process management to complete measures such as monitoring, alerting, and reporting within a predetermined or prescribed time cycle in the face of an outbreak of epidemic disease.

Two hospitals in China were selected for interviews as part of this study, focusing on the four sample events illustrated in Figure 3. Both hospitals had prior experience in the prevention and control of four infectious diseases, which aligned with the research objectives of our study. The interview outline was designed to address the research question of analyzing the causes of failure in direct reporting systems while incorporating the value realization theory of information systems. In-depth interviews were conducted at the two hospitals in January and June 2020; a total of 37 interviews spanning roughly 20 h were completed (see Table 1 for details). The interview procedure was standardized to ensure the data quality and usefulness. The interviews were held in mid-2020. Due to COVID-19 restrictions, most interviews were performed via telephone. To solicit comprehensive information, the interviews covered interviewees in various departments, including Infection department, other departments, prevention and protection section, and management. Each interview was performed by a single researcher, and the methodology was semi-structured, with questions based on the interview outline and follow-up questions based on the interviewees’ responses and views to elicit more complete and in-depth information. Each interview was audio-recorded. To ensure the adequacy of the interview data and material, interview highlights were collated shortly after each interview, and the two researchers discussed the material internally to flag any inaccuracies, which would be improved and supplemented during follow-up interviews.

**Table 1:** Descriptive statistics of the interviewees in the two hospitals.

Local-level hospitals	Number of interviews	Numbers and affiliations of subjects		Length (hours)
Grade IIA hospital (α)	1	Infection department	4	2.17
		Other departments	7	3.89
		Prevention and Protection Section	2	0.85
		Management	5	2.44
Total	1	18		9.35
Grade IIIA hospital (β)	1	Infection department	5	3.04
		Other departments	6	3.52
		Prevention and Protection Section	3	1.49
		Management	5	2.71
Total	1	19		10.76
Total	2	37		20.11

The research team sent a letter to each hospital prior to the interviews, which read as follows:

Dear XX Hospital,

Hello!

In order to gain a deeper understanding of the existing risk prevention and control system for major public health emergencies in China, and to develop a more reasonable and proactive response strategy, XXX has initiated and implemented personal interviews with hospitals as individual units.

These interviews will cover the responsibilities of your healthcare and management staff in the Prevention and Protection Section, Information Section, and Management Section, as well as the related workflow and personal responses to health incidents. If any of your personnel feel that certain interview questions infringe upon their privacy or are unwilling to answer, they can raise their concerns at any point during the interview. We will skip those questions and move on to the next. Additionally, your personnel have the right to request termination of the interview at any time. We fully understand and respect your decision in this regard.

Please be assured that your responses will be used solely for interview analysis by the researcher. Your organization and personal information will be treated with the utmost confidentiality, and all data will be handled anonymously to ensure the complete protection of your organization’s privacy.

Thank you very much for your organization’s participation!

Best regards,

XXX

The following is a concise outline of the interview:

1. In your opinion, how would you assess the preventive and control measures for COVID-19 at your facility? Are there any areas of improvement or challenges?
2. Has your facility established a system specifically designed to prevent and control outbreaks of infectious diseases similar to COVID-19? Could you provide a brief overview of how this system operates?
3. Does your facility have process management guidelines in place for the utilization of this system? If so, could you briefly describe these guidelines?
4. What are the responsibilities and actions expected from your department in terms of participating in the prevention and control of COVID-19? How do you currently fulfill these duties?
5. Do you believe that the rights and obligations of your department align effectively?
6. Have you encountered any situations where you were unable to carry out your duties or express your opinions regarding the prevention and control of COVID-19?
7. In your view, what measures do you believe should be taken in the future to enhance the prevention and control of infectious diseases like COVID-19?

In addition to telephone interviews, pertinent data were gathered from government agencies and relevant departments such as the Chinese Center for Disease Control; information on industry sales and business characteristics was collected from medical industry associations; and mass media data were collected from platforms such as social media and websites (see Table 2 and Supplementary Appendix for details). The inclusion of data from diverse sources increases the likelihood that the data will complement each another and can be cross-validated to increase the sample’s validity (54). In summary, this study relied on telephone interviews and several data sources to assure the sufficiency, quality, and relevance of the data.

**Table 2:** Table of data sources and event types.

Data source	Event type	Number
National Health Commission and other relevant guidelines, policies, decrees, economic bulletins, statistical bulletins, etc.	Ebola hemorrhagic fever	31
	Middle East respiratory syndrome	14
	H7N9 avian influenza A	56
	COVID-19	63
Information from social media reports, websites, etc.	Ebola hemorrhagic fever	55
	Middle East respiratory syndrome	43
	H7N9 avian influenza A	79
	COVID-19	87
Information from industry associations on industry sales, business characteristics, trends, etc.	Ebola hemorrhagic fever	7
	Middle East respiratory syndrome	1
	H7N9 avian influenza A	13
	COVID-19	6
Public information, such as libraries and professional databases	Ebola hemorrhagic fever	1
	Middle East respiratory syndrome	3
	H7N9 avian influenza A	4
	COVID-19	7

#### 3.1.2. Data coding and analysis

Data were coded and analyzed in three stages: reduction, presentation, and conclusion and validation. Data reduction entails the selection and simplification of qualitative textual information, the construction of coding tables, and the conversion of data to codes. Presentation entails a comparative analysis of the reduced data coding tables to establish initial connections between the theoretical findings and case data. Conclusion and validation entail the formulation of a theoretical framework supported by data, as well as the ideas for changes to prevention methods that arise from the framework.

The data were gathered into textual documents and classified using known case study procedures. The classified documents were inspected and tagged, and content analysis was used to analyze significant events. The key events were manually coded with the user’s description and attribution of the key event. Back-to-back coding occurred separately, with team members collaborating prior to coding to establish the coding scheme, which included ideas, critical procedures, and the basic logic that connected concepts. The primary processes were as follows. First, the data were coded at a source level to identify the characteristics of the four risk types. Second, failure and non-failure events were subjected to collective coding using an information collation index to form a secondary library of entries on unknown risks, the inability to consult, system selection, system filling, too many layers, different calibers, data silos, early warning dissemination, social notification, resource allocation, the centralization of authority, single study and judgment, the independence of prevention and control, hierarchical reporting, and training practices. Third, the results of the secondary coding were coded at three levels according to the initial diagnosis, reporting system selection, system data reporting, early warning notification, organizational centralization, expert judgment, and organizational practice. These stages yielded the coding sources and data classification shown in Table 3.

**Table 3:** Table of coding sources and data classification.

Data sources	Data classification	Codification			
		Ebola hemorrhagic fever	H7N9 avian influenza A	Middle East respiratory syndrome	COVID-19
First-hand data	Information obtained through in-depth interviews	A1	B1	C1	D1
	Information obtained through informal interviews	A2	B2	C2	D2
Second-hand data	Relevant guidelines, policies, decrees, economic bulletins, and statistical bulletins of the National Health Council	a1	b1	c1	d1
	Information on sales, business characteristics, and industry trends published and maintained by industry associations	a2	b2	c2	d2
	Information obtained through social media reports and websites	a3	b3	c3	d3
	Public information obtained from local libraries, etc.	a4	b4	c4	d4

This article follows the qualitative research approach of ensuring credibility and validity by analyzing the criteria from four perspectives to control and evaluate the data collection and analysis: construct validity, intrinsic validity, extrinsic validity, and reliability.

Correlational features were found to exist between risk classes, prevention and control systems, and process management reactions to failure events (or comparable descriptions of failures) during the early examination of the textual material. The largest proportion of failure events, 83.62%, was identified among COVID-19 events, and this value was much greater than those for the other three types of risk class events (see Table 4). The dysfunctional events alluded to in Table 4 are the statistical frequencies of event reactions spanning the entire pandemics and clearly demonstrate proclivities for dysfunctional and non-dysfunctional behavior. To establish independence, statistical analysis was applied to the primary event reported in secondary sources if two events were addressed in the same article.

**Table 4:** Frequency of failure and non-failure responses to critical events for four types of risk.

	Failure events		Non-failure events		Total	
	Number	% of	Number	% of	Number	% of
Ebola hemorrhagic fever	11	10.48%	94	89.52%	105	100%
Middle East respiratory syndrome	7	10.29%	61	89.71%	68	100%
H7N9 avian influenza A	34	21.38%	125	80.50%	159	100%
COVID-19	229	83.62%	37	16.37%	266	100%

Due to the unusually high proportion of events describing failures, this paper separately codes the key event causes underlying the failures and non-failures of the direct reporting system and its prevention and control system in response to emergent risks for the COVID-19 pandemic, an unborn-unknown risk security event.

### 3.2. Simulation analysis

A determination of the value of prevention and control system modification necessitates a retrospective review of the degree to which the failure of such systems and process management systems to react to a pandemic disease outbreak would jeopardize public safety. In this study, the described situation is examined through a retrospective simulation utilizing available data on the Wuhan and national outbreaks in China, along with a basic sensitivity test. The focus is on modeling the effects of alterations in the direct reporting system and process management on the Hubei provincial and national outbreaks. It investigates how changes in the direct reporting system and processes impact the timing of closure measures and subsequently influence the progression and control of the outbreak.

**Figure 4:**
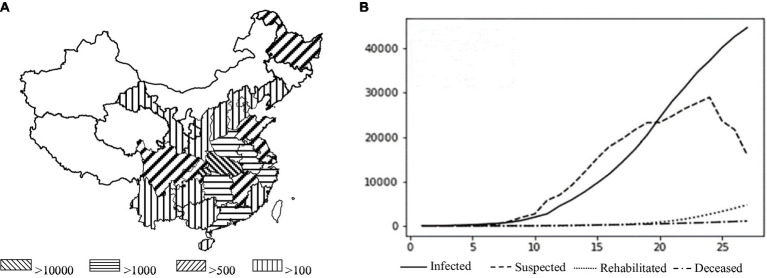
**(A)** Geographical distribution of the national epidemic (Source: the Chinese Center for Disease Control and Prevention). **(B)** Actual national outbreak data from January 15 to February 12, 2020 (Source: the Chinese Center for Disease Control and Prevention).

Epidemics of infectious illnesses capable of interpersonal transmission have become increasingly dangerous due to the rapid expansion of transportation networks and substantial increases in the ease of travel. Since the first case of pneumonia with an unknown etiology was reported in Wuhan on December 8, 2019, the number of infections associated with the COVID-19 pandemic has increased at a breakneck pace. As of December 1, 2021, the total numbers of confirmed cases and related fatalities worldwide were 127,398 and 5,697, respectively, and cases had been confirmed in all 34 provinces, municipalities, and autonomous areas in China. In Hubei province (excepting the Shennongjia Forestry District), all 12 prefecture-level cities, one autonomous prefecture, and three county-level cities under provincial control have enacted city closure measures, as shown in Figure 4A. Figure 4B depicts the Chinese national data during the initial outbreak from January 15 to February 12, 2020, providing a visual representation of the widespread transmission of an epidemic outbreak.

The Susceptible Infectious Recovered Model (SIR) model is a foundational model for studying the pace, geographical extent, and pathways of transmission of an infectious illness, and variations such as the Susceptible Infectious Recovered Susceptible (SIRS) and Susceptible Exposed Infectious Recovered (SEIR) models have been developed. Given the latent nature of SARS-CoV-2 infection, the SEIR model provides a more accurate description than the other models (55). Therefore, the SEIR model was used in this study to investigate the spread of SARS-CoV-2. The simulations were implemented using MTALAB software.

The simulation analysis consists of three steps. Firstly, this study simulated the number of confirmed cases in Wuhan, taking into account the actual situation of the city (e.g., quarantine and lockdown policies), and conducted a coincident analysis with the real situation to confirm the feasibility and validity of this simulation model. Secondly, it applies a C-SEIR model that includes governmental quarantine measures (C) and simulates the possible confirmed cases by changing the implementation date of the city closure measures. A comparative analysis with retrospective results was conducted to show that the implementation of government quarantine measures can be accelerated by changing the direct reporting system of infectious diseases. Lastly, it carried out a simulation of the implementation of loosely coupled process management to assess the effectiveness of the method in improving the timeliness of the government response.

The settings of the SEIR model are as follows: S (Susceptible) represents a healthy person lacking immunocompetence and susceptible to infection after contact with an infected person; E (Exposed) refers to a person who has been in contact with an infected individual but is not yet infectious; I (Infectious) refers to a patient with an infectious condition that can transmit the disease to susceptible individuals, turning them into E or I; R (Recovered) represents a person who has recovered from the illness and is immune. In the case of a lifelong immune infectious disease, recovered individuals may not be re-transmitted to S, E, or I. In summary, susceptible individuals become exposed when they have effective contact with an infected person, exposed individuals become infected after an average incubation period, infected individuals can recover and become immune, and recovered individuals are immune for life and no longer susceptible.

In the first simulation, we selected 2% of the total household population in Wuhan, as the initial vulnerable population base. The initial values of each parameter in the model are as follows: the number of contacts of infected people (*r*) = 21; the probability of infection for susceptible individuals (*b*) = 0.048; the probability of illness for exposed individuals (*a*) = 0.13; the number of contacts for exposed individuals (*r*^2^) = 21; the transmission probability for exposed individuals (*b*^2^) = 0.048; recovery probability (*y*) = 0.04; time (*T*) = *i* [for *i* in the range (0, 160)]. The transmission period began on December 30, 2019, when Li Wenliang and other physicians published seven verified cases on social media, leading to Wuhan’s implementation of a city closure on January 23. We calculated the number of people in each of the four groups on each day based on an iterative formula.

In the second simulation, we considered the effect of government measure C by adding the parameters: the newly diagnosed number of people with an exponential growth rate (*r*0) of 0.16 and the basic contagion number (R0) of 3.1. The highest number of confirmed cases in Wuhan was anticipated to reach 53,768, peaking on the 39th day after the emergence of SARS-CoV-2 when considering the actual scenario of city closure and quarantine. The change in the number of diagnosed people nationwide was simulated, assuming *T* is two days ahead and two days behind the actual date of the city closure. It’s important to note that the primary objective of this article is to establish an environment for analyzing the focal issue, rather than assessing the precision of this prediction. Figure 4 presents the actual situations of geographic distribution of the epidemic and the numbers of infected and recovered cases in China.

In the third simulation, we considered the impact of process management and the timeliness of government response on the number of confirmed cases. More details are provided in Section 5.4.

## 4. Result analysis

### 4.1. Result of simulation analysis

**Figure 5:**
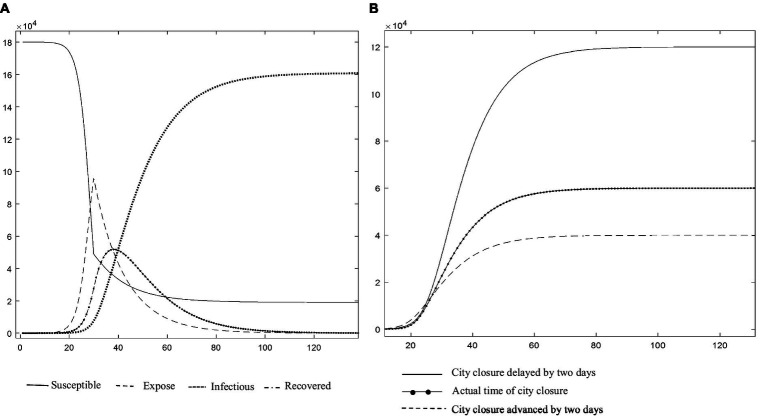
**(A)** Changes in the infection rate curves for China (Source: authors’ calculations). **(B)** Description of the impact of implementing city closure measures at different points from January 30 to February 7, 2020 (Source: authors’ calculations).

The major objective of a control system is to allow a quick reaction through real-time epidemic monitoring, transmission, and analysis, with the goal of treating patients with untreated or primary illness rather than those with severe illness. The quarantine measures implemented in Wuhan and numerous other locations during the COVID-19 pandemic were shown to effectively “treat the serious illness.” We applied the C-SEIR model, which includes government quarantine measures (C), and stimulated the national COVID-19 situation in China (e.g., Figure 5A). The basic reproducibility coefficient (R0) of SARS-CoV-2 was found to vary over time and is depicted in Figure 5A as having a realistic fit to the SARS-CoV-2 transmission curve. In sensitivity experiments, the date of implementation of the city closure measures was changed. No significant linear association was found between an earlier or later closure and the transmission inflection point; however, this analysis did reveal a significant correlation of the closure date with the number of confirmed cases. As seen in Figure 5B, advancing the closure by 2 days was associated with an estimated reduction in the cumulative number of infections by nearly a third, while delaying it by another 2 days would have doubled the cumulative number of infections. As posited by Wu et al. (56) and others, the number of infections was estimated to theoretically exceed 200,000 (R0 = 3.1) if effective prevention and control measures had not been taken during the early stages of the outbreak.

The above retrospective simulations of measures for the treatment of serious illness, such as city closures, at various points in time demonstrate that the introduction of these measures at various points had significant effects on the number of infections and the control of infectious disease spread. However, the risks to life, high socioeconomic expenses, and reduced quality of life of the population yielded two results that fell short of the management systems’ goals. Thus, this paper addresses the question of how to avoid the scenario depicted in Figure 4 and mitigate the severity of the scenario depicted in Figure 5 by transforming the prevention and control system into a multi-stage hierarchical warning and response mechanism with different risk control actions at various points in time. Thus, Figures 4, 5 serve as the baseline for an ongoing study that includes a comparative static analysis of the improvement options.

### 4.2. Results of case study

The analysis of case data in this paper reveals two major reasons for risk prevention and control failures. First, when the prevention and control system was applied, the data were primarily based on manual declarations and a lack of risk communication functions. Second, in process management, the organizational practices were null and void, the decision-makers were disconnected from the COVID-19 epidemic data, and epidemiology was over-centralized.

#### 4.2.1. Analysis of the key issues in the application of the prevention system

##### 4.2.1.1. Heavy reliance of data on manual reporting

As previously stated, the existing prevention and control system is managed locally and hierarchically, and the response model is based on the comprehensive entry of instances of danger by local health facilities or CDCs, as stated by an employee of a Shanghai hospital’s Infection Control Department (ICD): “Regardless of whatever department’s physicians detect an epidemic illness, they must complete an epidemic disease report card and submit it to the hospital’s Prevention and Protection Department, which will forward it to the appropriate authorities. The current national epidemic illness report card is based on ICD-10 diagnostic codes and contains information on 39 identified epidemic diseases. Most current hospitals and local disease control centers transmit fundamental data through manual reporting, while some hospitals link to the epidemic illness system via an Application Programming Interface (API) interface. Other data sources and functionalities only serve to supplement analyses or aid decisions and cannot serve as a trigger for illness reporting. Completion by individuals is subject to opportunistic motivations such as under-reporting by the person making the entry and cannot exclude subjective bias (57). Clearly, the data structure, which is dependent on individual completion, does not enable the system to respond agilely to unforeseen risk shocks.

Data standardization is required for automated reporting and analysis to be successful. Standardization at the data level refers to the use of uniform statistical techniques, procedures, and calibrations across data-collecting entities (58) to guarantee the generalizability of data and the efficacy of centralized analysis. In this study, the analysis of significant events revealed that variations in data quality and statistical content occurred across various healthcare facilities in the system’s reporting process. Using the COVID-19 prevention and control process as an example, small healthcare facilities and local communities concentrated on screening potential patients and identifying key observation targets when implementing the system and collected only personal information, whereas fever clinics required detailed symptoms to establish suspicion about individual cases. These differences in the purpose and scope of data gathering between units resulted in a lack of data sharing.

“Consistency in the statistical caliber, the quickness with which an unknown illness may get a correct diagnosis, and the ease with which patients can obtain [a diagnosis] all have a significant influence on statistics. And COVID-19 fails to address this issue.” Data standardization clearly has a direct influence on the prevention and control of an epidemic outbreak. For example, the COVID-19 outbreak prevention and control system included a “pneumonia self-reporting” feature; however, missing unique personal identifiers, such as ID numbers, resulted in the passive redundancy of a considerable quantity of data due to its rarity. As another example, a deficiency in the collection of crucial epidemiological history data adds to the difficulty of diagnosing and treating individual patients. Obviously, in medical diagnostics, the availability of vast volumes of unstructured data precludes the instant exploitation of the value of these data (6). A typical example from existing control systems is the direct reporting of pneumonia unspecified (PUE), which relies on both symptom descriptions entered in natural language and standard indicators such as the body temperature and white blood cell and lymphocyte counts, which require manual processing before being integrated into the database and thereby reduce the amount of data collected. Such non-standard language must be manually processed prior to integration into the database, thus lowering the data use efficiency.

##### 4.2.1.2. Lack of a risk communication function

The massive spread of an unexpected epidemic illness not only jeopardizes people’s health but also creates widespread fear throughout a society. Information on widespread diseases may be promptly transmitted via borderless information exchange routes on the Internet, such as between official media outlets, self-published media outlets, and the public.

However, the current prevention and control system is a data-driven information system that connects medical institutions with the CDC; it lacks a response function for risk communication to the general public. Official information on COVID prevention and control has frequently been communicated through press conferences or public notifications issued by the Health and Welfare Commission, whereas interactive communications between public entities regarding the COVID-19 risks in different regions, population flow tracking, personal health guidance, hotspot tracking, real information and disinformation, and other related topics have primarily been transmitted by Ding Xiang Doctor, Baidu, and other Internet platforms. As a result, there has been a dearth of broad information exchange between the government and the people, as exemplified by the following: “Article 18 of the Communicable Disease Prevention and Control Law specifies the CDC’s detailed responsibilities but does not specify whether the CDC has the authority to release information about epidemics as opposed to direct disposal,” and “I, as a local government, do not have the authority to communicate information about epidemics.” The legislation and remarks from government officials are ambiguous and indicate a dearth of government services tasked with informing the public about hazards.

A situation wherein the public does not have adequate access to official information and alternative channels (e.g., self-published media) continue to publish “false news” or “rumors” inevitably will result in a great deal of unconscious behavior among the public, making it more difficult to prevent and control risks such as mass shootings, the exodus of inhabitants from areas like Wuhan, and the frenzy over mask purchases. We proceeded to host the dinner this year as we deemed the epidemic’s propagation to be restricted to person-to-person, and so did not have adequate notice. This vividly shows how the absence of risk communication channels between the government and the public may contribute to the escalation of an unexpected epidemic crisis and result in additional harmful outbreaks. Accordingly, this article argues that creating effective risk communication channels between the government and the public is crucial for coping with unanticipated risk shocks.

Table 5 summarizes the textual analysis and provides typical instances of referenced evidence of failures in response to risk shocks at the level of prevention and control systems.

**Table 5:** Classification of prevention and control system failure events and examples of evidence.

Dimension	Main constructs	Coding entries	Examples of evidence (typical citation)
Choose and complete the reporting system (45)	System selection (44)	12	“Direct reporting of PUEs is one of the CDC’s tasks and is activated by specific situations.” (d3) “Prior to January 20, new coronavirus pneumonia was not recognized as a ‘legal infectious illness’ and hence could not be utilized for the national Web-based prevention and control direct reporting system’s 2-h direct reporting window.” (d3) “Normally, we avoid the direct reporting system, especially if we are unsure whether or not it is an epidemic, and the system will not allow us to enter the PUE.” (D1)
	System reports (23)		“The direct reporting method is complicated, and many physicians are unfamiliar with how to use it.” (d3) “If you are unable to use the direct reporting method, you will be sent to the hospital’s prevention and protection department.” (D1) “Although I’m in the prevention and protection section, I’m actually an administrator, and it’s especially difficult to fill in epidemics that are not in the system.” (D1) “The communication of suspected patients is still done by telephone to inform the health committee and the CDC.” (d3)
System data reporting (53)	Excessive layers (17)	21	“There are many levels of data reporting, including national, provincial, municipal, and district, and there is a procedure for data reporting that begins with the agency responsible for epidemic disease surveys at the district level.” (d3)
	Numerous calibers (31)		“Epidemiological statistics are also highly reliant on two critical variables: the statistical quality and the diagnostic technique. The consistency of statistical calibration, the capacity to rapidly identify the correct diagnosis for an unknown condition, and the access of patients to a prompt diagnosis may all have a significant effect on statistics. This is where the reaction to COVID-19 fell short.” (d3)
	Silos of data (14)		“The majority of hospitals no longer communicate patient data in a timely and synchronized manner. This results in hospitals grossly underestimating the devastation and effect of unknown diseases when they strike, and it is detrimental to bottom-up oversight of decision-making and growth within organizations.” (d3)
Early warning bulletin (54)	Early warning issuance (31)	22	“This year’s continuance of the Vanguard Banquet was based on our earlier assessment that the spread of this virus was restricted to human-to-human transmission, implying that there was inadequate notice of the incident.” (d3)
	Social bulletin (46)		“While Article 18 of the Communicable Disease Control Law delineates the CDC’s precise tasks, it does not provide the CDC the authority to release information on epidemic illnesses with direct disposal authority.” (d4)
	Resource allocation (19)		“Patients must be diagnosed using nucleic acid testing, and the shortage of nucleic acid testing reagents in the previous period resulted in some delays in risk prevention and management.” (d3)

The response frequencies in the “Main constructs” column are shown in brackets.

Table 5 and the preceding analysis and findings demonstrate that non-automatic data generation and the non-standardized formation of data structures at multiple levels have made it difficult for the existing prevention and control system to achieve a true, timely, accurate, and complete presentation of data during the COVID-19 risk prevention and control process, and have made it even more difficult to share and apply information efficiently. This disjointed and uneven approach to data gathering, sharing, and processing has impeded the prevention and control process, making it harder to react to unforeseeable risk shocks. Simultaneously, this data structure has made it more difficult for the prevention and control system to serve as a platform for critical risk communication to the public, hence lowering the impact of government information sources on society. Thus, development efforts should be directed toward making the data structure systematically responsive to unforeseen risk shocks and allowing continuous and high-frequency risk communication between the government and the public after a sudden pandemic outbreak.

#### 4.2.2. Analyses of critical concerns in process management

##### 4.2.2.1. Process practices are deficient

A prevention system connects epidemic disease surveillance efforts with informational exchanges between health and disease control administrations at the commune, district, municipal, provincial, and national levels, enabling an agile systemic patterned response to structured data on born-known or unborn-known risks. Due to the temporal latency associated with data propagation, nimble model responses may also be developed. However, for unborn-unknown epidemic diseases, the reporting process of the prevention and control system is highly susceptible to routine formality, as expressed by a CDC system physician during an interview: “It has not happened [in epidemic diseases] in so many years that medical institutions have generally not taken it selves.” Due to such evolution and solidification of practices via repetition, epidemic disease underreporting, late reporting, misreporting, and underreporting continue to occur even when the prevention and control system is equipped with technical functions such as data storage, structured form entry, data analysis, and standardized report presentation (30).

Fearing that the incorrect assessment of an unborn-unknown risk may result in overreaction and negative consequences, such as a social panic and economic decline, local hospitals and even CDCs tend to take the most cautious approach possible by avoiding responsibility as much as possible and adhering to the traditional practice of reporting at each level, centralized aggregation, and centralized research. As one clinician at Wuhan Zhong Nan Hospital stated, “Previously, clinicians reported unexplained pneumonia to the director, who then reported to the director, who then reported upward.” In other words, a hospital would use the system to report at the municipal or provincial level rather than the national level.

Additionally, a clinical judgment manifests as a diagnosis requiring further research, which the computer system cannot identify immediately and hence cannot report directly. Taken together, the above information shows that the current process management system is deficient in terms of effective organizational practices that enable rapid information exchange and efficient decision support in response to unforeseeable risk shocks. Furthermore, the existing division of authority and responsibility, organizational practices, and other factors have precluded the effective application of information systems.

##### 4.2.2.2. Disconnected frontline decision-makers

Theoretically, given the widespread effects of disease outbreaks with unknown risks, the process management system’s local response sensitivity is crucial to the success of early prevention and control efforts. However, the development of local early response sensitivity in process management systems has raised concerns about procedural legitimacy, including the rights to make decisions about outbreak epidemics, to diagnose and characterize diseases, and to disseminate information.

Currently, only the national CDC has the authority to diagnose and characterize illnesses. During the early stage of the COVID-19 pandemic, clinical detection of the causative factors (e.g., mycoplasma, bacteria, viruses) using imaging in combination with conventional methods, such as viral nucleic acid testing of blood samples, during diagnosis and treatment had not yet been determined; accordingly, process management required centralized reporting to local CDCs and health committees. At the micro level, while the causative agents of epidemic outbreaks with unknown risks exhibit characteristics that distinguish them from previously identified causative agents, information about these agents’ potential for harm, transmission routes, viral characteristics, and effective treatments is uncertain, thus hindering prevention and control systems’ ability to develop plans. For instance, the nucleic acid test kit for SARS-CoV-2 was not available until 76 days after the first confirmed case. In another example, SARS-CoV-2 was not discovered as the seventh to infect people until it had already caused a substantial epidemic.

Simultaneously, standardized epidemic reporting using deterministic indicators within the prevention and control system might create uncertainty for non-frontline healthcare staff reacting to an outbreak (59). This uncertainty, combined with the fact that the incubation time for SARS-CoV-2 varies by individual, caused the first clustering of COVID-19 outbreaks to be poorly defined, which complicated centralized reporting and decision-making. For instance, the first universally generalized history of exposure to South China seafood markets in the context of unknown epidemic illnesses associated with feverish symptoms and positive viral testing revealed a huge number of hidden dangers during later prevention and management efforts. The previously discussed procedural legality concerns have led departments such as the Health and Wellness Commission to passively choose a strategy wherein decisions are deferred in favor of conducting research. As a staff member responsible for epidemic disease reporting card review at the Wuhan Jiang Han District CDC stated in an interview, “While the reporting of sudden epidemic diseases is bottom-up, these new diseases must first be reported.” Considering the rapid and widespread transmission of an unexpected epidemic disease, this reaction technique objectively has become a component of the rapid amplification of unborn-unknown risks.

##### 4.2.2.3. High concentration of information power

Currently, only the government health agency can provide information about unexpected epidemic illnesses. According to the Prevention and Control of Infectious Diseases Law, the Emergency Response to Public Health Emergencies Regulations, and the Ministry of Health’s Information Release Program on Statutory Infectious Diseases and Public Health Emergencies, the State Council’s health administrative department is responsible for informing the public when an infectious disease outbreak or epidemic occurs. In response, a lawyer from the Beijing Jing Law Firm provided the following representative quote: “The current laws and regulations place an unreasonable burden on the publication of information on sudden epidemic diseases, which, combined with the lengthy and inefficient pre-reporting procedure, tends to delay information publication.” These statements imply that the concentration of all power for distributing information about a breakout epidemic within government health departments has made it impossible to react swiftly to the effects of endogenous unknown risks.

Table 6 summarizes typical instances of reported data on the management of risk processes that have resulted in failures to prevent and control unborn-unknown risks.

**Table 6:** Examples of evidence of prevention system failure at the process management level.

Dimension	Main constructs	Coding entries	Examples of evidence (typical citation)
Organizational centralization (37)	Concentration of power (37)	31	“Due to the complexity of characterizing big epidemic outbreaks, after the data model has identified the infectious virus, openness and open access are often not in the hands of local governments, and choices are made by the National Center for Disease Control (NCDC). Following the receipt of the data, the NCDC is required to send specialists to verify and validate the data. The whole process may be significantly delayed, which is detrimental to the prompt disclosure, prevention, and control of hazardous new major epidemic disease strains.” (d3)
Expert assertion (33)	Single study (27)	32	“Detection of a sudden significant epidemic, of course, necessitates waiting for a professional consultation, but that is much too difficult at present; everyone is busy, and it is still mostly up to one expert.” (D1)
	Immunization independence (14)		“Our outbreak of major epidemic disease prevention and control is all about minding our own business; how can we possibly care about others? It’s impossible to handle difficulties with multi-sectoral communication; there’s a chasm between the operational and administrative sectors.” (D1)
Organizational practices (21)	Layers of reporting (19)	33	“The decision-making behavior and administrative procedures necessary for several levels of approval are expensive to society, time-consuming, and may not follow the normal pattern of abrupt significant epidemic development.” (d3) “Without substantial external action, a newly recognized breakout of a severe epidemic, such as (novel coronavirus pneumonia), may only increase in lockstep with the threat’s severity.” (3d)
	Training practices (21)		“The surveillance reporting process is too complicated, necessitating cascading, sample surveillance, and so on, and the pressure to report and process is intense. Additionally, this system was supposed to monitor SARS and human avian influenza, which have not occurred (sudden major pandemic disease) in so many years that medical institutions have generally ignored them, even though the CDC also conducts annual training for subordinate CDC staff and hospitals, which is almost always a walk in the park.” (d3)

From Table 6 and the study findings, it can be inferred that the centralization of the authority to diagnose and characterize illness and distribute information within the CDC and government health departments has resulted in a closely integrated and centralized decision-making system. Although a benefit of this decision-making structure is that the components of the prevention and control system are tightly coupled and work cooperatively (60), the drawback is that the sensitivity of local frontline healthcare professionals is diminished. Although the intuitive judgments made by these professionals are not based on substantial research, they serve as a locally sensitive early warning method for the control system. Thus, the path of changes in management should be determined by the ability to integrate democratic and centralized decision-making, and by the ability to combine consistent action in the prevention and control system with improved sensitivity in local responses.

## 5. Improvements and verification

Based on the analysis and findings presented above, a conclusion can be drawn that increasing the local sensitivity of data, establishing risk communication channels, implementing adaptive process management changes, and establishing a loosely coupled decision-making structure are three areas for improvement intended to help the prevention and control system cope with unborn-unknown risk shocks.

### 5.1. Triangulated validated risk communication system

As mentioned previously, the absence of a response function for risk communication in the prevention and control system means that the government frequently has lacked sufficient information sources for studying and making decisions about sudden major epidemics with unknown risks; in addition, the public often struggles to obtain effective information protection, which frequently has resulted in the escalation of negative situations. The purpose of change is to expand the application models of existing prevention and control systems to address the lack of existing prevention and control systems and risk communication functions in the population. Such expansion should include the development of risk warning models, risk control models, and risk communication models. In the event of a large-scale epidemic involving unknown risks, the prevention and control system should shift from management to risk communication mode by, for example, providing a transparent information sharing channel between medical institutions and the public to avoid widespread panic caused by rumors and speculation, or by providing public information for guidance and official actions. In another example, online psychological assistance could be provided to individuals to prepare them for early warnings and changes due to unexpected risk shocks.

**Figure 6:**
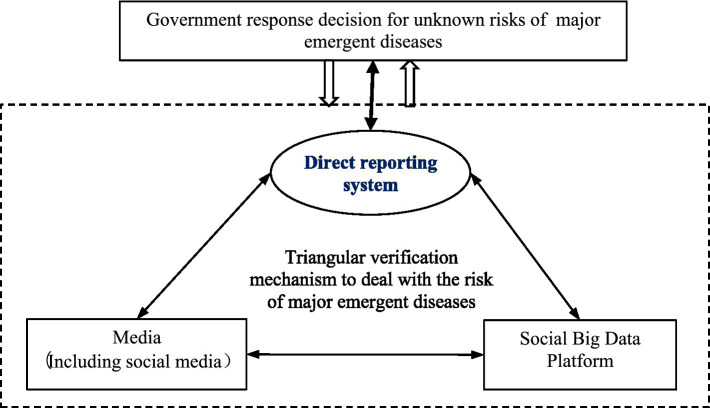
Triangulated validated risk communication system (Source: authors’ summary).

This application is referred to as a triangulated risk communication system in this study. This triangulated risk communication paradigm has been verified and can differentiate between an epidemic alert, warning, and reaction. An alert is a source of early warning information, and alerts and reactions are connected but not synonymous. Specifically, big data platforms and official media outlets serve separately as “social whistle blowers,” while the prevention and control system serves as an “internal whistle blower,” thus constituting a triangulated verification system. Accordingly, the prevention and control system can more effectively communicate the risk of a major epidemic and provide sufficient information to guide the government’s response decisions, while considering multiple objectives such as political, economic, and diplomatic considerations (see Figure 6).

Unconfirmed news is widely disseminated via channels such as social media platforms, in which the truth is likely to be distorted, leading to unconscious group behavior (26). For example, misinformation or true/false seeking information about a sudden major epidemic disease garners 41% public attention and 35% retweets (26). Figure 6 disinformation drew considerable attention. The validation of risk misinformation is classified in Figure 6 as risk over-prompting validation, risk under-prompting validation, and unprompted risk validation. The purpose of over-prompting verification is to ensure that the stated risk is accurate and not exaggerated. The purpose of under-prompting verification is to ensure that the stated risk is accurate and that the degree of risk is fully described in accordance with the facts. The purpose of unprompted verification is to ensure that the stated risk is accurate and not exaggerated. In terms of rumor detection and insight, artificial intelligence (AI) can be used to efficiently and rapidly evaluate whether a piece of material is a headline, incorrect information, or rumor by aggregating and analyzing data on sources, sensitive words, and authors (61, 62). Throughout the COVID-19 outbreak, Tencent Medical Dictionary and Ding Xiang Yuan used data mining and sentiment analysis to monitor and disprove hot rumors, providing over 100 million individuals with expert responses and official facts. AI-based risk warning verification may substantially enhance verification speeds and accuracy. As AI applications gain traction and as prevention and control systems, media systems, and social big data platforms develop AI models for mutual information verification, this triangular verification mechanism is expected to yield a systematic and agile mode of response to the impacts of unborn-unknown risks.

Figure 6 implies that all subjects communicated via the triangular risk alert system can be independently self-validated, followed by triangular validation of the required information and, eventually, a risk warning created via full analysis. During the self-validation stage, the initial information source is identified, and the publisher’s qualifications are determined by analyzing the user’s professional field, registration time, activity pattern, and other portraits; the credibility of the information is calculated by summing the proportions of people with various attitudes, their respective levels of credibility, and other information. Next, all information sources within the subject are analyzed. After self-validation is complete, the information moves into the triangular validation stage, where the “social whistleblower” and “internal whistleblower” are assigned to distinct risk alert topics, and all risk alerts are subject to a risk warning by the prevention and control system. After completing the triangulation of danger alarms, the system executes the associated risk alert. Thus, the triangulation process shown in Figure 6 will enhance the CDC’s ability to issue early warnings about major epidemics while providing the government with strong information support to minimize uncertainty in making predictions and increase the sensitivity of reactions to major epidemics.

### 5.2. Change of data structure in the prevention system

Although a triangulation verification system can address the issue of risk communication, the application of the prevention and control system relies heavily on manual declarations, which is substantially impeded by the system’s lack of risk communication functionality. With the application of digital technologies such as 5G networks, big data, and AI, the prevention and control system’s data structure can be further optimized to improve information monitoring, data exchange, and multi-dimensional analysis capabilities in response to unforeseeable risk shocks. While it remains impossible to predict with certainty when and where a major epidemic disease with unborn-unknown risks will occur, it is possible to remove some of the uncertainties and transform them into manageable risks during the process of bearing risk shocks, thereby transforming passivity into activity, by increasing the local sensitivity of data and establishing an agile, big data- and AI-powered system for the initial transmission paths and regional unknown risks. As such, the purpose of this study is to change the prevention and control system’s core automated data gathering function to a risk communication function that uses AI and big data analysis technologies.

One path forward may involve connecting hospital information systems (HIS) above the township level to the direct reporting system to achieve the underlying data; this would include the automatic collection, exchange, and storage of basic data such as test results, electronic medical records, and clinicians’ diagnostic reports of suspected patients in epidemic disease departments. AI technologies then can extract the data for full analysis, detect aberrant values, and prompt the reporting of possible dangers, therefore displacing the data production system that depends heavily on spontaneous reporting by physicians before triggering alarms. Changes in data sharing between HIS and direct reporting systems may begin at third-tier hospitals in provincial capitals, followed by progressive expansion to medical institutions at all levels, including townships and counties. Simultaneously, healthcare personnel generate a considerable quantity of unstructured data when diagnosing cases of infectious illness, and thus it is difficult to totally remove the use of manual entry. In this regard, the direction of change involves introducing blockchain technology based on big data to establish a private chain within China’s disease control system, connect each hospital system to a blockchain node, and synchronously enter personal reporting information, HIS, electronic medical records, test reports, and other contents into the blockchain.

As a second path forward, we advocate for adjusting data standardization in three areas of the prevention and control system: method standardization, process standardization, and caliber standardization. Method standardization involves creating and implementing a set of uniform report card formats across all healthcare institutions to standardize and unify the electronic medical record paradigm used by each hospital and thus improve data structuring. Extracting more commonalities from sudden major epidemics, incorporating these into a unified questioning framework, and using techniques such as deep learning in AI to improve the efficiency of natural language processing could reduce the cost of data collection. Process standardization can reduce errors in the underlying data and the impacts of complex environments on an organization (50). For example, variations in examination procedures and the questioning of suspected patients may introduce systematic bias at the lowest levels of the underlying data process. By using structured big data to construct data calibrations, we can ensure that data from disparate sources can be legitimately included in an aggregate analysis.

### 5.3. Creation of loosely coupled adaptive process management

**Figure 7:**
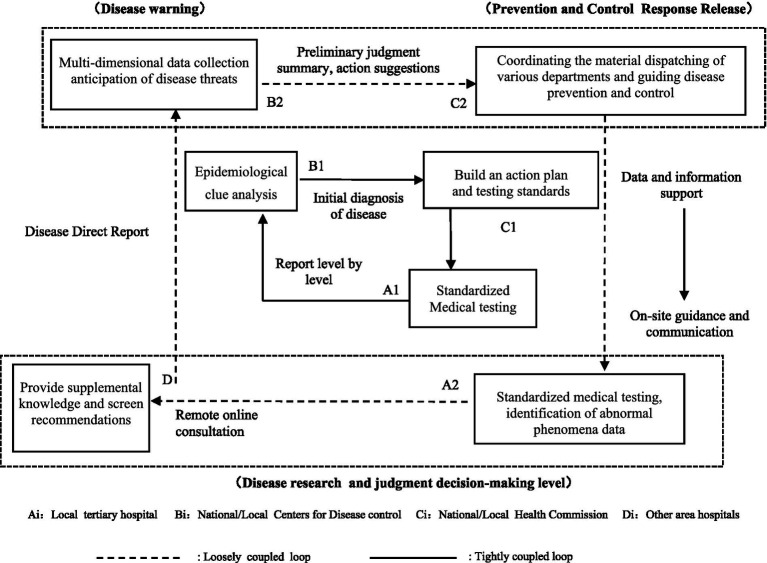
Main architecture of loosely coupled processes for adaptive process management change (Source: authors’ summary).

The objective of a change in adaptive process management is to ensure an organic combination of democracy and centralization of research and decision-making authority, as well as the combination of consistent action by the prevention and control system and improved sensitivity of local responses. Adaptive process management, i.e., changes in loosely coupled process management, can be used to deal with the high level of uncertainty associated with the initial phase of an outbreak of an unknown major epidemic disease and to address how the direct reporting system’s response to unknown risks evolves into organizational inertia (see Figure 7).

Figure 6 illustrates a sample architecture of loosely coupled processes based on tandem IT, with an emphasis on management that fosters complementarity between collective and individual decision making, thereby establishing an organic union of democracy and centralization in the study and analysis of unborn-unknown risks and associated decision-making. The inner cycle in Figure 7 represents the operational flow of a tightly integrated prevention and control system during risk responses and the generation of synergies and responses to born or unborn known hazards and born-unknown risk shocks. Synergies and responses are established when reacting to unborn-unknown risks that have not yet been created by compensating for the absence of a tightly connected inner circle with a loosely coupled outside cycle composed of A2–B2–C2–D based on tandem IT, as shown in Figure 7. The inner and outer cycles of the main process during the adaptive process management of the prevention and control system aim to avoid the rigidity of processes and practices associated with a single coupling pattern within the system, as well as degradation of the system caused by decentralization (63), by combining consistency of action within the prevention and control system with increased sensitivity to local responses.

Process optimization can be viewed through three lenses to produce complementary communal and individual decision-making in the outer circle, thus establishing the democratic centralization of research, analysis, and decision-making regarding unborn-unknown risks. First, epidemiological judgment is completed via remote consultations with representative medical institutions and CDCs in the region to avoid bias in judgments and analyses caused by regional differences in experience and the limitations of individuals’ knowledge (64). If irreconcilable differences in the assessment of the epidemic arise, other healthcare providers may be asked to conduct studies and analyses, but the final judgment of the local healthcare provider is retained. Simultaneously, information on individual cases is shared transparently in real time via the direct reporting system between local tertiary hospitals and the National Health Commission, as directed by authorized management. Second, frontline healthcare workers typically collect a large amount of information that is difficult to present in a structured manner in the short term, and medical diagnostic capacity is limited at the township level. Third, to prevent local governments from engaging in opportunistic behavior, such as the selective reporting or whitewashing of material for various reasons, it is essential to evaluate the legislation governing public health crises. The Emergency Response to Public Health Emergencies Regulations and the Measures for the Administration of Information Reporting on Public Health Emergencies and Infectious Diseases have optimized information publication by, for example, granting the authority to publish information on epidemic disease warnings (e.g., the Meteorological Bureau’s disaster warnings) to the CDC in collaboration with the government and the Health and Welfare Commission. Thus, the government has retained the competency in multi-objective decision-making needed to face unknown risks, while the CDC has acquired the loosely linked reaction capabilities necessary to cope with unknown risk shocks.

Thus, four possible outcomes can be predicted from the combination of CDC warnings and the government’s response to a major unanticipated risk: accurate CDC warning and timely government response, accurate CDC warning and untimely government response, false CDC alarm and timely government response, and false CDC alarm and untimely government response. Ideally, the CDC would issue an accurate warning and the government would respond promptly; in the second-best scenario, the CDC would issue a false alarm and the government would respond prematurely. The other two eventualities must be assessed on a case-by-case basis. As with weather predictions, which are not always accurate or timely, precise pandemic warnings and false alarms coexist in this loosely coupled management process. Adaptive process management strategies for dealing with unknown risk shocks also include modifying how epidemic warnings are perceived and building a loosely connected system for managing public opinion regarding an epidemic.

### 5.4. Simulation analysis of changes in prevention system

Will the three modifications outlined above result in improvements? In the following sections, data on the COVID-19 pandemic presented in Figures 4, 5 are used as a baseline for simulating the implementation of improvement strategies to assess the effectiveness of measures intended to change the prevention and control of unborn-unknown risks with respect to response timeliness. The simulations are based on the SEIR model, which approximates reality by considering the asymptomatic post-infection latency period (i.e., exposure) in the population. To address the effect of unborn-unknown hazards, a multi-stage, hierarchical system of warnings and government reactions is established according to the separation of information warnings and the prevention and control response distribution authority as suggested in the preceding management change approach. First, based on the R0 deduced by Cole et al. (18), Wu et al. (56) and others, the R0 for a new epidemic is assumed to be unknown information during the early period of an unknown risk shock. Second, based on the studies of Wu et al. (56) and Cai et al. (57) additional parameters such as the number of initial infections, contacts of infected people within a population, transmission Both risk communications from the CDC and the government’s reactions, such as the dissemination of warning materials and travel prohibitions, can significantly reduce the frequency of interpersonal exposure in the community.

**Figure 8:**
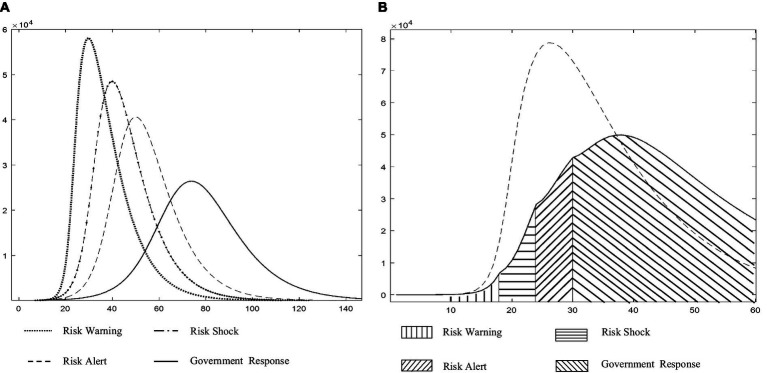
**(A)** Comparison of the impact on the epidemic of releasing information and imposing bans at the same time (Source: authors’ calculations). **(B)** Phased release of information and imposition of bans on the impact of the epidemic, phased versus reality (Source: authors’ calculations).

Figure 8A illustrates the effect on epidemic illness transmission of a travel restriction that is issued or adopted at time *T* = 7, according to the SEIR model. In the figure, the vertical coordinates indicate the numbers of infected patients. The tighter the restrictions on human contact, the more effective the overall outbreak control, assuming that the patients are not re-infected after recovery and the epidemic transmission area is closed. When the interpersonal contact restriction is set to three people (i.e., fellow household members), the peak number of infected patients is less than half of what it would be when the risk is present. This finding shows that, first, the CDC’s risk warnings and the government’s response actions must complement one another. This finding is consistent with empirical research in the literature (35, 65) that considers risk warnings and governments’ response actions to be information systems and management processes, respectively. Second, when dealing with unknown risk shocks, separating epidemic disease risk alerts from the issuance of government prevention and control responses results in more timely responses than are achieved when these measures are combined. The former is a loosely coupled decision-making framework that incorporates expert judgment and multi-objective decision-making.

During the early phases of an unknown risk shock, decision-making is hindered most by the paradox of overreaction and lagging response. An unborn-unknown risk is considered to be of unpredictable intensity, with the potential for quick and extensive spread. To achieve epidemic control while avoiding a social panic and economic downturn due to an overreaction, an improved strategy based on the information presented in Figures 6, 7 must assume that different information sharing, risk communication, and action measures will be implemented during the four early stages of an emerging epidemic by various entities such as local tertiary hospitals, the CDC, and the National Health Commission. In the first stage, risk alerting occurs at *T* = 7, i.e., when an unknown disease emerges and non-infectiousness cannot be ruled out; at this point, the population makes its own reference and travel planning decisions. In the second stage, if the rate of suspected case generation continues to rise for more than a week, the CDC is required to issue a risk alert and social risk communications even if the suspected epidemic remains undiagnosed. This stage can be considered as the strategic command center. After one week, if the growth rate of the epidemic is not accompanied by a decrease in population travel, the CDC increases its risk warning. This third phase may be considered as the tactical command center. In the fourth stage, if the number of individuals infected with the unknown disease continues to increase, the government reaction must intervene to avert a significant epidemic even if the disease has not yet been fully described and identified. This is the period during which danger is contained. During this stage, the prevention and control system switches to risk communication mode, wherein professional medical institutions such as the CDC take actions ranging from risk level assessment to risk downgrading and risk removal, and provide professional assistance to the government in making decisions on the resumption of work, mass production, and other aspects. Undoubtedly, a change in any of the aforementioned variables may result in an appropriate upgrade or downgrade of the risk warning and response level to avoid an overreaction related to epidemic prevention and control.

Figure 8B illustrates a simulation of a COVID-19 outbreak according to the four stages discussed above. The results in Figure 8B demonstrate that while the overall development of an epidemic cannot be reversed during first three phases beyond temporarily slowing the increase in patient numbers, theoretically, the multi-stage, hierarchical early warning and government response mechanism based on the improved strategies proposed in Figures 6, 7 can substantially reduce the peak number of infections in comparison with the real scenario, while gaining valuable time for disease diagnosis, analysis, and decision-making. These gains would enable a society to develop a short-term prevention and control plan for unknown risk shocks. According to the findings in Figure 8B, a multi-stage, hierarchical warning and response mechanism is a manifestation of the idea of integrating the governance, and is a potential direction for exploring the reform of risk prevention and control measures in response to sudden major epidemics.

## 6. Discussion and conclusion

### 6.1. Main conclusion

Ultimately, the aspiration of all nations is sustainable development. Epidemic prevention and control serve as a critical orthodoxy of sustainable health development, offering a strategic bulwark for the continuance of economic and social growth. Success in this domain can catalyze further supportive resources for diversified sustainable development arenas. Hence, epidemic prevention and control hold a pivotal position in the sphere of sustainable health development.

Through simulations and case studies, this article examines the successes and failures of a prevention and control system in responding to four types of risk shocks and analyzes the primary reasons for the system’s failure to respond to unborn-unknown risk shocks in terms of its data structure and management processes. First, the data structure and supporting management procedures of the prevention and control system are nimble and adaptable to known and unknown risks, but the system inability to adapt to new risks was found to be the primary reason for its failure to deal with the COVID-19 pandemic. Second, to ensure that the prevention and control system remains agile and adaptive in the face of an unknown risk shock, its data structure should be generated automatically using underlying data from healthcare institutions nationwide and a private blockchain, and a triangularly validated risk communication system should be established. The management process should be proactive and adaptive (i.e., a loosely coupled decision-making process). Third, the establishment of a good loosely coupled decision-making framework to address unborn-unknown risk shocks does not imply that the current tightly linked decision-making structure should be disregarded or abandoned. The closely connected structure remains relevant for known risks that have not yet shown themselves, as well as for unknown risks that have manifested themselves, and is a regular decision-making structure in risk prevention and control management.

### 6.2. Creative contributions

This article introduces the concept of loosely coupled adaptive process management and explores the theory of adaptive management systems built around a loosely coupled process and a triangulated validated risk communication architecture to address two critical scientific issues: how to deal with the complementarity of collective and individual decision-making in the context of a disease epidemic, and how to define the fuzzy boundary between adequate prevention and overreaction. Accordingly, it provides sound advice to advance sustainable development from the perspective of the prevention and control of pandemic outbreaks.

First, this article proposes complementary theories of tightly and loosely coupled decision-making architectures via adaptive prevention and control management within the 2 × 2 category framework of born-known risks, unborn-known risks, born-unknown risks, and unborn-unknown risks, elucidates the primary reasons why the prevention and control system failed to function as expected when faced with the shock of COVID-19, and theoretically revealing the realization mechanism.

Second, a multi-stage, hierarchical warning and government response mechanism, including a risk alert, decentralized decision-making, a strategic control window involving risk warning and communication, a tactical control window involving risk alert escalation and enhanced communication, and risk proliferation containment and government action responses, is proposed by drawing on the concept of integrating the governance of untreated, primary, and serious diseases. Theoretically, the operational mechanism for responding to major public health events is explained, and the article expands on the theoretical approach to defining the blurred line between adequate prevention and overreaction in the field of prevention and emergency management through the study and assessment of major public health risks and related decision-making.

Third, the integration of the theory of information systems value realization into the study of changes in prevention and control systems intended to cope with the unknown risks posed by major disease outbreaks has established complementarity between the application of information systems and changes in the management processes of prevention and control systems. This demonstrates that it is possible for multiple actors to implement prevention and control measures by interacting and collaborating with each other even in the context of a national public health crisis (34). That is, the principle of complementarity between information systems and management processes holds and enriches the context of research on the value of information systems.

### 6.3. Limitations and further research

The main limitations of this study are threefold. First, it was difficult to conduct in-depth face-to-face interviews due to the COVID-19 pandemic; instead, this research relied on telephone interviews, which have limitations that are difficult to circumvent, and a few direct interviews with CDC administrators. Second, the chain-of-evidence analysis mainly focused on a case study of the failures to respond to an unborn-unknown risk, with little discussion of the variables underlying failures to respond to risks of the other three types. Third, this study mainly explored problems in the management of epidemic outbreak prevention and control systems and processes in the Chinese context (66) and proposed relevant solutions, whereas the situations in other countries were not researched. Taken together, the first two limitations have no substantial impact on the stability of the results and policy recommendations in this paper. Future research should focus on conducting in-depth interviews with personnel from various management sectors to identify complementarities and synergies between local governments, healthcare commissioners, CDCs, and healthcare providers. In addition, epidemic outbreak prevention and control systems and process management in other countries should be explored, and experimental comparisons should be made to ensure that research on global sustainable health development is up to date.

### 6.4. Policy recommendations

Additionally, this article sets forth the following policy suggestions for advancing prevention and control management paradigms via innovation.

First, strategic needs for reforming and upgrading the disease prevention and control system should be met, including implementation of the prevention gateway and promotion of deep integration of the Internet, big data, artificial intelligence, and direct reporting systems. Recommendations: (1) The prevention and control system should integrate seamlessly with electronic medical record systems, HIS, laboratory information management systems, medical image archiving and communication systems, radiology information systems, and other systems at all levels of hospitals across countries and should standardize the API interfacing ports. Blockchain technology should be used at all levels, beginning with community hospitals, to create a private blockchain for the CDC system. Thus, the diagnosis and treatment process of frontline physicians will involve the automatic collection of disease information and thus truly create underlying data that are tamper-proof and traceable from bottom to top and horizontally throughout the prevention and control system, establishing a platform and set of data for the complete visualization and hierarchical authorization of data sharing systems from the local to the central level. (2) A standardized report card format should be created for clinicians to use and apply concurrently from top to bottom. In addition, top-down deployment to hospitals should be synchronized, and a query language database for self-consultation by patients should be developed rapidly to lower the cost of information exchange. (3) An early warning mode and a switchable prevention and control mode should be added to the current prevention and control system’s application mode. (4) The current system’s information sharing channels should be extended, with communication channels between official healthcare institutions and the general public, and the channels should be enabled to perform various application scenario tasks in a variety of modes. To accomplish these recommended changes, the prevention and control system must undertake specific technical work in three areas: expanding data generation methods to diversify the data source channels, promoting data standardization to increase the possibility and richness of data sharing, and establishing a risk communication channel to improve information symmetry between the government and society.

Second, comprehensive integration of the Internet, big data, AI, and public health management systems should be advocated. An adaptive process management system for responding to sudden major epidemics should be established, and an organic balance between democracy and centralization in the authority over decisions regarding research and judgment should be promoted along with an organic balance of consistent action within the prevention and control process with increasingly sensitive local responses. Recommendations: (1) Empowerment of the prevention and control system with the ability to warn and notify about hazards should be considered. The Health Care Commission’s risk warning authority should be transferred to local tertiary institutions, with initial judgments based on information from frontline healthcare professionals. When the initial judgments are discordant, the prevention and control system may be utilized to facilitate data exchange between several hospitals and consultation with distant experts while maintaining the ultimate judgment of the local healthcare institution. For instance, the networked CDC and local medical institutions should be tasked with creating an organic balance between democracy and centralization in significant risk research, assessment, and decision-making, which would systematically enhance the capacity to study, evaluate, and make risk-related choices. (2) The public health management system’s multi-stage and hierarchical early warning and response mechanism should be established and enhanced. The CDC is responsible for risk distribution and communication at three levels, namely danger alert, and risk warning, while the government determines whether and how to react using measures such as restriction, quarantine, and city closure. At various phases, the risk alert and risk response levels are correlated differently, and the CDC risk alerts and government action responses function in a complementary manner to aid in risk prevention and management. (3) A loosely connected decision-making system should be created to address the effects of unforeseeable future hazards. A big data, AI-driven agile response model of the initial transmission paths and regional risks of unborn-unknown risks should be developed to provide powerful risk information for joint prevention and control mechanisms at the central and local levels, as well as decision support for the government in delineating the fuzzy boundary between adequate prevention and overreaction and alleviating the pressure of limited rationality in government.

Third, the CDC’s professional management should be strengthened to enable it to handle tasks such as expanding the prevention measures, boosting risk awareness, and enhancing its capacity to investigate, appraise, and make decisions about important hazards. This article recommends strengthening the CDC’s professional management capacity in three areas: (1) adoption of a personnel management system that balances mobility and stability and establishment of a system of transparency and procedures and a system of rotation for the primary person in charge (e.g., rotation from one location to another every 3 years and promotion from one location to another); (2) strengthening of the funding guarantee mechanism, improving the rate of funding allocation, directing funds toward improvement of the prevention and control system, and promoting organizational reform in the prevention and control of epidemic diseases; and (3) at the legal level and procedures to establish a mechanism for budgeting and allocating special funds, and arranging for an independent body to budget and control funds and disclose financial information in a timely manner.

Fourth, preventive management science and effective synergies between public health and medical services should be established and developed, and prevention and control, joint prevention and control, mass prevention and treatment, and other adaptive preventive management knowledge systems should be integrated effectively. Although emergency management science is reasonably well established, the theoretical community lacks a concept of preventive management and a relevant knowledge structure. Preventive management is needed for reforming and strengthening disease prevention and control systems and appropriate risk emergency response mechanisms in terms of disciplinary development, knowledge systems, and education and training. Recommendations: (1) a sub-discipline of preventive management should be established on par with emergency management in a disciplinary management system. Preventive management is a new multidisciplinary field of study that focuses on the theory and practice of adaptive preventive management. Its basic competencies include preventive medicine, systems engineering, information systems management, and public health management. Preventive management focuses on achieving an organic balance between democracy and centralization in terms of the authority to make decisions regarding preventive research and judgment, as well as an organic balance of consistent action in the prevention and control system with increased local response sensitivity. Research in this area addresses the management theory of preventive research and judgment, assessment, and decision-making in response to the impacts of major public security events. (2) Prevention management concepts and knowledge systems should be integrated into the national public health emergency management knowledge system; prevention management research teams, academic groups, and scientific research groups should be established and developed; the declaration and establishment of prevention management topics should be encouraged and promoted; and the exploration of frontier theories in prevention management should be encouraged. (3) The integration of theoretical research into prevention management should be promoted. Simultaneously, preventive management should be included in fundamental knowledge training provided to general practitioners, frontline medical and nursing staff, and others.

## Author’s note

1. Bain & Company, “Beating the Epidemic, Grateful to Move Forward - Is New Crown Pneumonia Trapping the Chinese Economy? Report, 8 February 2020, Sohu.com, https://www.sohu.com/a/371485904_282725.
2. Bluedot Canada issued its first alert for COVID-19 on 31 December 2019 with the help of an AI model, nearly a week ahead of the US Centers for Disease Control and Prevention’s (CDC) alert on 6 January 2020.
3. Even though the scientific community disagrees on the true origin of COVID-19 based on genetic data analysis, the first confirmed COVID-19 patient in Wuhan and the first widespread spread of the COVID-19 epidemic, COVID-19 remains as an endogenous unknown risk in terms of the form in which the risk occurs.
4. Considering that the epidemic is still on-going, and the number of new confirmed, suspected, and fatal cases is still dynamic, this paper does not aim to give an accurate calculation of the number of people affected or economic losses.
5. The Prevention and Control of Infectious Diseases Law in Article 38, the Emergency Regulations for Public Health Emergencies in Article 25 and the Ministry of Health’s Information Dissemination Program for Statutory Infectious Diseases and Public Health Emergencies together specify that the State establishes an information dissemination system for emergencies. The competent health administrative department of the State Council is responsible for releasing information on emergencies to the public. If necessary, the competent health administrative departments of the people’s governments of provinces, autonomous regions, and municipalities directly under the central government may be “authorized” to release information on emergencies within their administrative regions.
6. Research Report on Public Awareness and Information Dissemination on Novel Coronavirus Pneumonia in 2020, 26 February 2020, China.org, http://www.chinanews.com/zwad/2020/02-26/8664390.html.

The variables, action processes, and strategy function settings of the simulation model in this paper are available upon request. Interested readers are encouraged to request this information directly from the authors. Ethics approval was obtained for the study.

## Data availability statement

The raw data supporting the conclusions of this article will be made available by the authors, without undue reservation.

## Ethics statement

Ethical review and approval was not required for the study on human participants in accordance with the local legislation and institutional requirements. Written informed consent from the participants was not required to participate in this study in accordance with the national legislation and the institutional requirements.

## Author contributions

YZ led and conceived the study, performed the literature review analysis, conducted the case study and simulation analysis, and prepared the manuscript. PF assisted with literature collection, analysis, and writing, and also contributed to the case analysis. JS revised the manuscript and secured funding for the project. YY provided valuable input during the manuscript revision process. All authors contributed to manuscript revision, read, and approved the submitted version.

## Footnotes

^1^The Sustainable Development Goals (SDGs). 2021. https://unstats.un.org/sdgs/report/2021/.
