# Supplementary material for: Addressing unforeseen public health risks via the use of sustainable system and process management
Source: Front Public Health. 2023 Nov 10;11:1249277. doi: 10.3389/fpubh.2023.1249277 (PMC10667458; doi:10.3389/fpubh.2023.1249277)
Supplement: Supplementary file 1 [file Table_1.docx]

**Appendix**

| Data sources | Specific data sources |
| --- | --- |
| Information obtained through social media reports, websites | Baidu News, 26 February 2020, http://baozha.net/a/baoguang/2020/0226/278030.html |
|  | Tencent News, 31 January 2020, https://wxn.qq.com/cmsid/20200131A0EK3T00 |
|  | YeoGuide, 1 February 2020, https://www.iyiou.com/p/122846.html |
|  | Tencent News, 1 February 2020, https://wxn.qq.com/cmsid/20200201A06U1A00 |
|  | Tencent News, 25 February 2020, https://xw.qq.com/cmsid/20200225A0N9JF00 |
|  | Sina Finance, 4 February 2020, https://baijiahao.baidu.com/s?id=1657578290093409520&wfr=spider&for=pc |
|  | Cady Network, 27 February 2020, http://club.kdnet.net/dispbbs.asp?id=13612083&boardid=1 |
|  | China Economic Weekly, 1 February 2020, https://baijiahao.baidu.com/s?id=1657347317979276659&wfr=spider&for=pc |
|  | Sina Technology, 31 January 2020, https://baijiahao.baidu.com/s?id=1657237199289839873&wfr=spider&for=pc |
|  | QQ.com, 27 January 2020, https://new.qq.com/omn/20200127/20200127A06YMO00.html |
|  | Global Times, 22 January 2020, https://baijiahao.baidu.com/s?id=1656389836692015264&wfr=spider&for=pc |
|  | Sohu.com, 22 January 2020, https://www.sohu.com/a/368348341_115362 |
|  | QQ.com, 18 February 2020, https://www.sohu.com/a/368348341_115362 |
|  | Phoenix, 18 February 2020, http://news.ifeng.com/c/7uAtjaf28QF |
|  | Sohu.com, 25 February 2020, https://www.sohu.com/a/375634299_120228 |
|  | Check Daily, 25 February 2020, http://newspaper.jcrb.com/2020/20200225/20200225_003/20200225_003_2.htm |
|  | Observer, 26 February 2020, https://baijiahao.baidu.com/s?id=1659552715715471148&wfr=spider&for=pc |
|  | Surf News, 27 January 2020, https://baijiahao.baidu.com/s?id=1656870559667879135&wfr=spider&for=pc |
|  | Oriental.com, 27 January 2020, http://news.eastday.com/eastday/13news/auto/news/society/20200127/u7ai9052467.html |
|  | Hexun.com, 4 February 2020, http://news.hexun.com/2020-02-04/200181836.html |
|  | Sina Technology, February 4, 2020, https://tech.sina.com.cn/roll/2020-02-04/doc-iimxyqvz0064649.shtml |
|  | Medsci, January 23, 2020, http://www.medsci.cn/article/show_article.do?id=627f1866952e |
|  | Surf News, 24 February 2020, https://www.sohu.com/a/375346260_260616 |
|  | Economic Observer, 3 February 2020, http://m.eeo.com.cn/2020/0203/375484.shtml |
|  | Economic Observer, 30 January 2020, http://www.eeo.com.cn/2020/0130/375237.shtml |
|  | Chuangzhan Health, 27 February 2020, http://www.chzhw.com/health/jk31499.html |
|  | China Legal Network, 27 February 2020, http://www.legaldaily.com.cn/government/content/2020-03/03/content_8133268.htm |
|  | Trongzhan Health, 27 February 2020, the  http://www.chzhw.com/health/jk31499.html |
| Relevant guidelines, policies, decrees, economic bulletins, statistical bulletins of the National Health Commission, CDC, etc. | Wuhan Municipal Health and Health Commission, official website, December 31, 2019, http://wjw.wh.gov.cn/front/web/showDetail/2019123108989 |
|  | Official website of the China Center for Disease Control and Prevention (CDC), accessed January 21, 2020, http://www.chinacdc.cn/jkzt/crb/zl/szkb_11803/jszl_11813/202001/t20200121_211327.html |
|  | Official website of the China Center for Disease Control and Prevention Control, 27 January 2020, http://www.chinacdc.cn/jkzt/crb/zl/szkb_11803/jszl_11811/202001/P020200127544648420736.pdf |
|  | Official website of the Chinese Center for Disease Control and Prevention (CDC), 9 September 2006, http://www.nhc.gov.cn/jkj/s3576/200901/f2e5f428cea64912ba7e93c2f13dabff.shtml |
|  | Official website of the Chinese Centre for Disease Control and Prevention (CDC), 20 January 2009 |
|  | http://www.nhc.gov.cn/jkj/s3578/201304/5e94f31ed8714fcf9af60b4e819b7686.shtml |
|  | Official website of the Chinese Centre for Disease Control and Prevention (CDC), 1 May 2009 |
|  | http://www.nhc.gov.cn/jkj/s7923/200904/4c8a365461204f3498fc13ac1230b899.shtml |
